# Further Evidence against a Momentum Explanation for IOR

**DOI:** 10.1371/journal.pone.0123666

**Published:** 2015-04-16

**Authors:** Jonathan W. Harris, Christopher D. Cowper-Smith, Raymond M. Klein, David A. Westwood

**Affiliations:** 1 Division of Kinesiology, School of Health and Human Performance, Faculty of Health Professions, Dalhousie University, Halifax, NS, Canada; 2 Department of Psychology and Neuroscience, Faculty of Science, Dalhousie University, Halifax, NS, Canada; State University of New York Downstate Medical Center, UNITED STATES

## Abstract

Reaction times to targets presented in the same location as a preceding cue are greater than those to targets presented opposite the cued location. This observation can be explained as a result of inhibition at the attended location (IOR), or as facilitation at the location opposite the cue (opposite facilitation effect or OFE). Past research has demonstrated that IOR is observed reliably, whereas OFE is observed only occasionally. The present series of four experiments allows us to determine whether or not OFE can be explained by eye movements as suggested by previous authors. Participants' eye movements were monitored as they were presented with an array of four placeholders aligned with the four cardinal axes. Exogenous cues and targets were presented successively. Participants (N=37) completed either: i.) cue-manual and cue-saccade experiments, ignoring the cue and then responding with a keypress or saccade, respectively, or ii.) manual-manual and saccade-saccade experiments, responding to both the cue and the target with a keypress or saccade respectively. Results demonstrated a reliable IOR effect in each of the four experiments (reaction time greater for same versus adjacent and opposite cue-target trials). None of the four experiments demonstrated evidence of an OFE (reaction times were not significantly lower for opposite versus adjacent cue-target trials). These results are inconsistent with a momentum-based account of cue-target task performance, and furthermore suggest that the OFE cannot be attributed to occasional eye movements to the cue and/or target in previous studies.

## Introduction

There is a reliable bias against responding to targets appearing at recently attended locations. In the first study to reveal this effect, Posner and Cohen [1] presented participants with three placeholder locations, one at central fixation and one to the left and right of fixation. Participants maintained eye position on the central fixation stimulus while an uninformative cue, return-to-centre signal, then target were presented in succession. Participants were instructed to ignore the cue, but to acknowledge the appearance of the target by pressing a single button. When the cue-target onset-asynchrony (CTOA) was less than 200 ms, reaction time (RT) was faster for targets at the cued location; however, when the CTOA was greater than 200 ms, this effect reversed and participants were relatively slower to respond to targets appearing at the cued location. Posner & colleagues [1,2] reasoned that the onset of the cue, despite being non-predictive of the future location of the target, briefly captured spatial attention and that after attention was disengaged from the cued location and re-engaged at the central location an inhibitory ‘tag’, viz inhibition of return (IOR), discouraged the return of attention to the cued location.

Before settling on an inhibition-based account, Posner and Cohen [1] considered—and ultimately rejected—several alternative mechanisms for the slower responding at the cued location. One of these, and the one that the present study allows us to explore, was that spatial attention may possess a quality much like the physical property of momentum, such that attention has a tendency to continue along an established line of motion. Were such an "attentional momentum" mechanism operating, the primary effect would be a reaction time benefit at the uncued location (which we will call the opposite facilitation effect, or OFE). According to this proposal there is nothing special about, and no explicit inhibitory ‘tag’ attached to, the location of the cue. Instead, there are benefits when the most recent direction in which attention had moved (from the cued location back to center) remains unchanged. Importantly, with only two possible target locations, one cannot distinguish between the inhibitory tagging and attentional momentum accounts, because both simply predict faster performance at the uncued location relative to the cued location.

**Fig 1:**
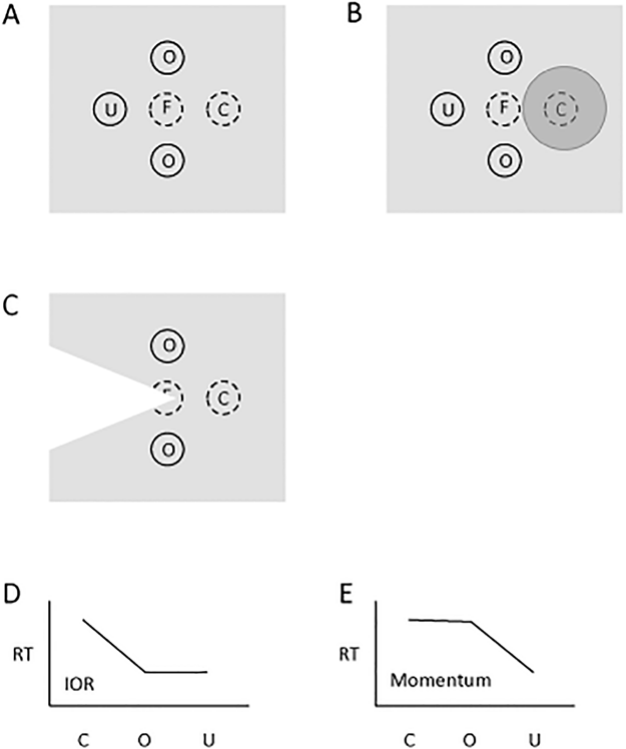
Experimental predictions. Representation of the experimental setup (a) and the predictions that are made for reaction time if IOR (b) or attentional momentum (c) are operating. a) Each trial begins with the participant fixating a stimulus at the center of the screen (marked as F). A cue is presented at any of the locations marked by the letters O (orthogonal), U (uncued) and C (cued). In this example the cue is presented to the right of fixation. At the time of the presentation of the target (at any of the 4 peripheral locations where cues could have been presented) it is assumed that attention, after having been captured by the cue has returned to fixation. b, c) Illustrated here are the hypothesized distributions of the effect of IOR (b) and attentional momentum (c) at the time of target presentation. In (b) the circular region, darker than the grey background, represents the inhibited region where RT to targets will be increased. Here a gradient centered on the cued location is assumed. In (c) the wedge-shaped region lighter than the grey background represents the facilitated region where RT to targets will be decreased. Here it is a direction (away from the originally cued direction) that is facilitated with a gradient of decreasing facilitation as the angular deviation of the target's direction from the direction of attentional momentum increases. d) Pattern of results predicted by the gradient of IOR illustrated in panel (b). e) Pattern of results (this will be referred to as the opposite facilitation effect, OFE) predicted by the gradient of attentional momentum illustrated in panel (c).

Posner and Cohen [1] expanded their experimental design to include four possible peripheral cue/target locations (left, right, up and down from centre) to permit a direct comparison between momentum and inhibition accounts, which make different predictions about response times for uncued target locations that are the same distance from fixation and equidistant from the cued location and location opposite the cued location (see Fig 1). We will refer to these uncued locations as "orthogonal". According to the inhibitory tagging account, RTs to uncued-orthogonal locations should be similar to those at the uncued-opposite location (Fig 1b and 1d), as the inhibitory tag is restricted to a gradient around the cued location. According to the opposite facilitation account, targets appearing at locations in the direction of the vector of attentional momentum should show a benefit relative to both the cued and uncued-orthogonal locations (Fig 1c and 1e). An intermediate pattern might be observed if both mechanisms were operating. Posner and Cohen’s [1] results (as described on p. 538 of their paper, conforms to the pattern displayed in Fig 1d favored the inhibitory tagging mechanism over the attentional momentum mechanism, as reaction times for targets appearing at the cued location were slower than to all uncued locations which did not differ from each other.

Despite the fact that attentional momentum had been proposed, tested and rejected by Posner & Cohen [1], the idea was re-advanced by Pratt et al. [3]. Later Spalek & Hammad [4] would suggest that Posner and Cohen’s failure to observe an OFE may have resulted from a lack of statistical power. Although the results of these studies seem to provide evidence in favor of momentum, subsequently Snyder and colleagues [5] re-analyzed the data from Pratt et al. [3] showing a lack of support and, in two separate studies [5,6], employing similar methods to Spalek & Hammad [4], but with a more detailed analysis they also failed to obtain a reliable OFE.

Snyder, Schmidt and Kingstone [6], for example, conducted a four-location cue-target manual response experiment in which participants were presented with a central placeholder and four peripheral placeholders, as illustrated in Fig 1a). Participants were presented with a peripheral cue followed by (1) a peripheral target, or (2) a return-to-centre cue and then a peripheral target. Participants were required to detect the appearance of the target by pressing a single key. Participants were instructed to maintain central fixation for the duration of the experiment, but compliance with this instruction could not be verified, as no eye tracking was used in the study. Omnibus analysis revealed significant OFE and IOR (though, notably, with IOR larger in magnitude than OFE for all target locations), however, when RT data were examined as a function of the initial cued location (i.e., up, down, left, or right), significant OFE was found only for cues occurring at the top and bottom of the display whereas IOR was present for each of the four cue locations. Because IOR was found for all locations, but OFE was not, the authors reasoned that the two effects must be independent and that a single AM mechanism was unable to account for the pattern of performance. The authors suggest that the sporadic occurrence of OFE may be “…an artifact of eye movements or idiosyncratic attentional strategies carried out by participants.”

The "eye movement" suggestion is made possible because, with few exceptions, past research examining attentional momentum has failed to monitor eye movements. Thus, as suggested by Snyder et al. [6] it is possible that the sporadically-obtained OFEs are somehow generated by occasional reflexive eye movements to the cue and/or target, despite the fact that participants were instructed not to look away from the central location. Machado and Rafal [7] provide a notable exception and some support for the Snyder et al. [6] suggestion. They tested for inhibition versus momentum using button press detection responses to a peripheral target while monitoring eye movements in a four-location cue-target task. In one condition, the cue was an arrow at fixation calling for a saccade in the indicated direction. In the other condition, the cue was a to-be-ignored brightening of one of the peripheral boxes. In both conditions, a cue-back at the location of the original fixation was used to encourage gaze (in the endogenous overt orienting condition) or attention (in the exogenous covert orienting condition) to return to center. In both conditions, Machado & Rafal [7] reported significant IOR (i.e., the cued location was slower than uncued-orthogonal locations) but no significant OFE (i.e., uncued-orthogonal locations were similar to the uncued-opposite location). Importantly, according to Snyder et al.'s [6] re-analysis of the data from this and all the other experiments they considered useful for testing for IOR versus attentional momentum, in Machado and Rafal [7] the relatively small OFE (4–5 ms compared to an average of 9–10 ms in the studies that failed to monitor eye position) was non-significant regardless of the location of the cue. This consistency of their failure to observe an OFE distinguishes Machado and Rafal's [7] findings from all other studies reviewed by Snyder et al. [6], each of which obtained a significant (albeit usually smaller than the IOR score) OFE effect on one, if not more than one, of the 4 or 8 axes tested.

The present experiment allows us to directly test Snyder et al.'s [6] suggestion that the sporadic findings of an OFE might be associated with eye movements to the peripheral cues, or targets. In two of the four experiments (E1 and E2), participants made saccades to a peripheral target following an uninformative peripheral cue. In one of these experiments (E1), participants were instructed to refrain from looking at the cue, but responded to target stimuli with a saccadic localization (cue-saccade). In the other experiment (E2), participants were explicitly required to look at the cue and then return their gaze to the central position before the target appeared; saccadic responses to the target were again required (saccade-saccade). In two parallel experiments (E3 and E4), participants refrained from making any eye movements and acknowledged the appearance of the peripheral target by pressing a single response key. In one of these experiments (E3) participants were instructed to ignore the peripheral cue (cue-manual), and in the second (E4) participants were to detect both the cue and the target (manual-manual). To our knowledge, there have been no previous studies of OFE in which participants were explicitly required to respond to the *peripheral* cues.

If the generation of attentional momentum depends on the execution of an eye movement to the cue stimulus and subsequently back to the central location (as suggested by Snyder et al. [5]), then we would expect to find evidence of its effect (i.e., OFE) in the saccade-saccade experiment but in none of the other experiments. If, however, attentional momentum is generated when attention shifts to the location of the cue and then back to centre, but can be revealed only for eye movements to subsequent targets, then we would expect to find an OFE in the cue-saccade and saccade-saccade experiments, but not in either of the manual response experiments because eye movements will have been discouraged and excluded from these data.

## Materials and Methods

### Participants

37 undergraduate students (13 Male and 24 Female) at Dalhousie University participated in the current study in exchange for course credit. All participants reported normal or corrected-to-normal vision.

### Ethics Statement

This project received ethical approval from the Dalhousie University Research Ethics Board (project number 2011–2589), which follows the standards laid out in the Tri-Council Policy Statement 2 (Canada). All participants provided informed written consent prior to participation.

### Materials

SR Research Experiment Builder (SREB) was used in combination with EyeLink II (SR Research Ltd., Mississauga, ON) eye tracking system to create and carry out this study. The flow of the experiment and eye movements of the participants were measured using the EyeLink II (SR Research Ltd., Mississauga, ON) head-mounted, video-based eye-tracking device (sampling rate = 500 Hz; spatial precision <0.01°; spatial accuracy <0.8° RMS error). Calibration of the EyeLink II was carried out in the same picture-plane used to display the experimental stimuli. EyeLink DataViewer software (SR Research Ltd., Mississauga, ON) digitizes the pupil in order to describe the location of the visual gaze fixations. Respondents’ eye movement data (saccadic reaction times) and key presses (manual reaction times) were recorded to a text file which was exported into an excel file and ultimately uploaded to SPSS v.15.0 for further statistical analysis.

### Procedure

The current study consisted of a four experiments: cue-saccade (E1), saccade-saccade (E2), cue-manual (E3) and manual-manual (E4) response. The same peripheral cue and target stimuli and procedure was used in each of the four experiments. What differed between experiments were the responses made to the cue (no response vs. response) and the nature of responses (manual detection vs. saccade); note that a response was always required to the target. Each participant was assigned to either cue-respond group (N = 19), where participants completed both cue-manual (E3) and cue-saccade (E1) experiments, or respond-respond group (N = 18), where participants completed both manual-manual (E4) and saccade-saccade (E2) experiments. The order of group assignment was randomized and the order in which participants completed experiments was counterbalanced.

For the duration of each experiment, participants were seated 57 cm from a computer monitor upon which all experimental stimuli were presented. Participants were presented with a central square and four peripheral squares, all equal in size (with dimensions of 3.57 degrees of visual angle). The peripheral squares were positioned 12.2 degrees from the center of the center square, one above and one below the horizontal meridian and one to the right and one to the left of the vertical meridian. Thus, the 5-square placeholder arrangement formed a “+”. Target dimensions and relative orientation remained the same for all trials.

**Fig 2:**
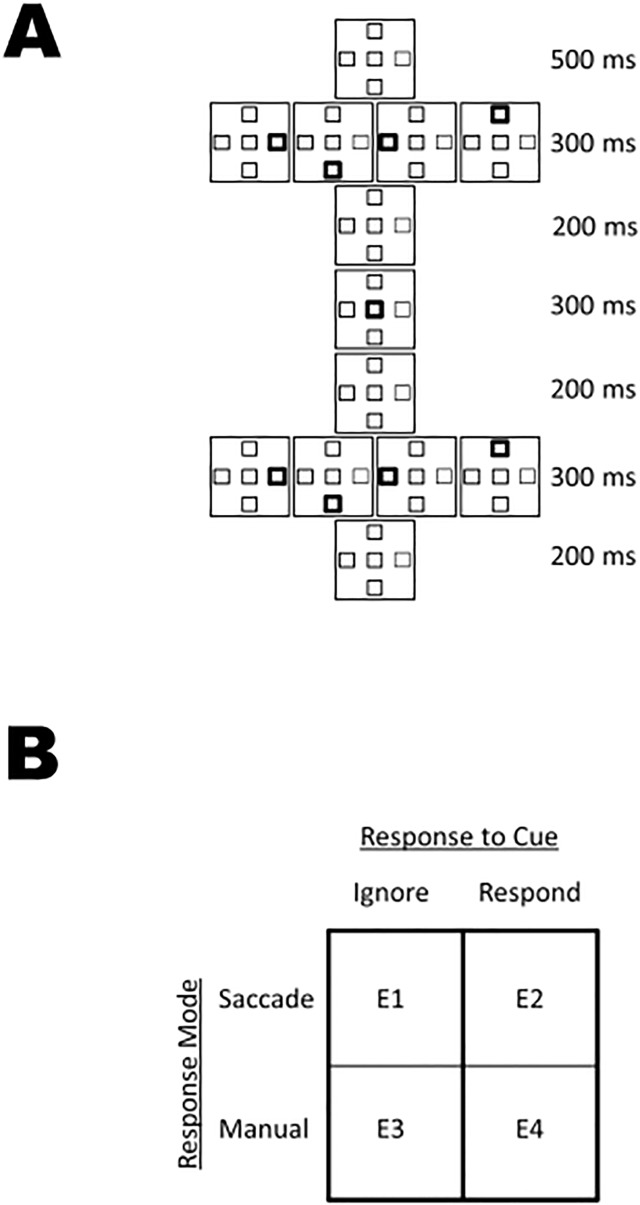
**Sequence of events in each experiment (a).** See text for explanation. **Conditions for Experiments 1–4 (b).** See text for explanation.

Each trial began with the presentation of a small circle at the centre of the screen to perform a drift correction procedure. Participants were required to fixate this central circle and press spacebar to initiate the trial. If the participant’s gaze was not located within 10.68 degrees of visual angle of the centre circle, a tone was presented, indicating that the participant should realign their gaze with the centre circle and press spacebar. This process continued until an acceptable calibration had been achieved. Participants were then presented with the four-placeholder array for 500 ms. Participants were instructed to maintain gaze at centre. Next, one of the four peripheral placeholders was bolded (to double its original line width) for 300 ms (Cue) after which it was restored to its usual line width for 200 ms (thus bringing the total duration of the “Cue” phase to 500 ms, as indicated in Fig 2A). In cue-manual (E3) and cue-saccade (E1) experiments, participants were required to ignore the cue and maintain gaze at centre. In manual-manual (E4) and saccade-saccade (E2) experiments, participants were required to respond to the cue by making manual detection (i.e., press a single key to acknowledge the onset of the stimulus) or saccadic responses (i.e., look at the cue), respectively.

Next, the central placeholder was bolded for 300 ms (Cue Back), after which it was restored to its usual line width for 200 ms (thus bringing the total duration of the “cue-back” phase to 500 ms, as indicated in Fig 2A). Participants were required to maintain fixation at centre (cue-manual, cue-saccade, manual-manual), or return gaze to centre (saccade-saccade).

Following the cue-back phase, one of the four peripheral placeholders was bolded for 300 ms (Target) after which its original line width was restored for 200 ms (thus bringing the total duration of the “Target” phase to 500 ms, as indicated in Fig 2A). Stimulus timing was not contingent on participant responding, so the cue-target onset asynchrony was 1000 ms. Participants responded to the onset of the target with a saccade (E1 and E2) or by pressing the spacebar (E3 and E4). The timing and nature of experimental stimuli is depicted in Fig 2A. In each experiment, all possible combinations of cue location (left, down, right or up) and target location (left, down, right or up) were presented, for a total of 16 possible trial types; all trial types were randomized and equally probable. Each of these combinations was presented 10 times, for a total of 160 experimental trials. To discourage anticipatory responding, catch trials (in which no target was presented) were added as ten percent of experimental trials, thus bringing the total number of trials to 176.

### Errors

Error trials were not recycled and were excluded from subsequent analysis. In the two manual response experiments (E3 and E4), participants were required to keep their gaze at the centre for the duration of each trial (failure to maintain gaze position within 10.68 degrees of the centre of the screen resulted in an error message: "You looked away from centre. 3000 ms penalty”). Although a circular region of interest with a diameter of 10.68 degrees might seem overly generous, the purpose was simply to exclude trials in which eye movements were made to targets during a required fixation period, or trials in which required saccades were made to incorrect targets. Given that targets were located 12.2 degrees away from central fixation, the regions surrounding fixation (5.34 degrees in any direction) and surrounding each target (5.34 degrees in any direction) did not overlap and therefore achieved the desired purpose. As noted by an anonymous reviewer, even if small eye movements had been made this would only strengthen our conclusion that opposite facilitation effects (OFE) are not associated with eye movements.

In E3, participants responded to the appearance of only the target (cue-manual), while in E4, participants responded to both the cue and the target (manual-manual) by pressing the spacebar. Any manual response made to the cue in the cue-manual condition resulted in the presentation of an error screen.

In the two saccade experiments (E1 and E2), participants made no manual responses. In E1, participants were required to saccade to the target but not the cue (cue-saccade) while in E2, participants saccaded to both the cue and the target (saccade-saccade). In the saccade-saccade experiment, participants moved their eyes back to centre after responding to the cue.

In each of the four experiments, failure to generate the required response to a target within 500 ms caused the trial to be aborted, and an error message to be displayed: “You failed to respond in time. 3000 ms penalty”. In the saccade experiments, correct responses were verified by checking whether the participant’s gaze was within 10.68 degrees of visual angle of the centre of the specified target location at the end of the allotted 500 ms period.

### Data Analysis

Manual reaction times were computed as the time difference between the onset of the target and the time at which the participant pressed the space key. Saccadic reaction times were computed as the time difference between the onset of the target and the time at which the participant initiated a saccade whose amplitude was greater than 2.0 degrees of visual angle. Reaction times from all error trials were excluded from further analysis, as were reaction times less than 100 ms (as these were assumed to result from participants anticipating the appearance of the target).

We analyzed RT data according to target location and the angular distance between the cue and target which will be referred to as "cue-target offset". Reaction time data from each of the four experiments were submitted to a 4 (target location; up, down, left, right) by 3 (cue-target offset; 0, 90, 180) repeated measures ANOVA. Data from -90 and 90 degree cue-target offsets were pooled in order to generate a single, 90-degree offset (orthogonal uncued) category. Pairwise comparisons were used to test for differences that directly assess IOR (i.e., RTs for 0 degree offsets are greater than for 90 degree offsets) and OFE (i.e., RTs for 180 degree offsets are less than 90 degree offsets). Post-hoc comparisons were used to explore effects due to target location. Mauchly’s test was used to test the assumption of sphericity. In cases where sphericity was violated, Greenhouse-Geisser corrected df, MSE and p-values are reported.

## Results

### E1: Cue-Saccade Condition

In the 4 (target location; down, left, right, up) by 3 (cue-target offset; 0, 90, 180) repeated measures ANOVA, there was a significant main effect of target location (F(3,54) = 6.8, MSE = 1678.94, p = 0.001): mean RTs for down, left, right, and up targets were 222, 192, 195, and 197 ms, respectively. Bonferroni-corrected pairwise comparisons indicated significant differences between down (222 ms) and left (192 ms) (p = 0.0055), down (222 ms) and right (195 ms) (p = 0.0023) and down (222 ms) and up (197 ms) (p = 0.0075). No other comparisons reached statistical significance.

**Fig 3:**
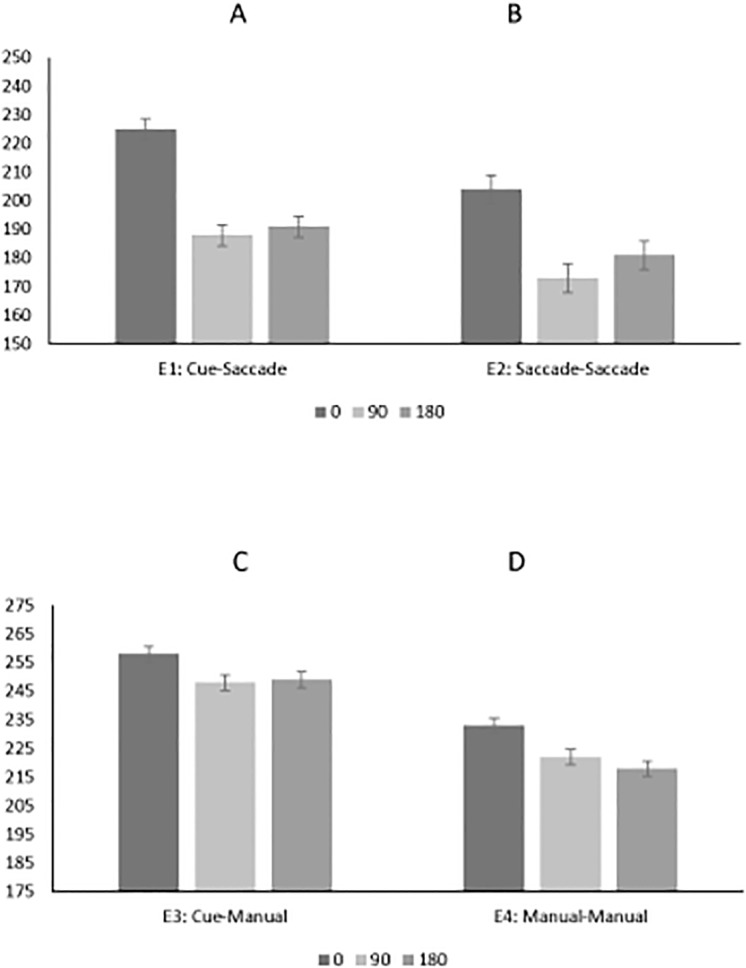
Saccadic reaction time (a,b) and manual reaction time (c,d) as a function of cue condition (cue-target offset). Data are shown for Experiment 1 (a), Experiment 2 (b), Experiment 3 (c) and Experiment 4 (d).

There was a significant main effect of cue-target offset (F(2,36) = 32.8, MSE = 963, p<0.001): mean RTs for 0, 90 and 180-degree offsets were 225, 188, and 191 ms, respectively. These data are presented in Fig 3a. Pairwise comparisons revealed significantly greater RT in the 0 degree (225 ms) compared to 90 degree offset conditions (188 ms; p<0.001) (IOR = 37 ms), while there was no significant difference in mean response times between the 90 degree (188 ms) and 180 degree cue-target offsets (191 ms; p = 1.0) (OFE = -3 ms). The interaction between target location and cue-target offset was not significant (F (3.82, 68.7) = 0.864, MSE = 1668, p = 0.486).

### E2: Saccade-Saccade Condition

In the 4 (target location; down, left, right, up) by 3 (cue-target offset; 0, 90, 180) repeated measures ANOVA, there was a significant main effect of target location on RT (F(1.95, 33.2) = 7.82, MSE = 1830, p = 0.002): mean RTs for down, left, right, and up targets were 206, 179, 180, and 181 ms, respectively. Bonferroni-corrected multiple comparisons indicated significant differences between down (206 ms) and left (179 ms) (p = 0.0017) and down (206 ms) and right (180 ms) (p<0.001). No other comparisons reached significance.

There was a significant main effect of cue-target offset (F(1.29, 21.9) = 16.4, MSE = 1792, p<0.001): mean RTs for 0, 90 and 180-degree offsets were 204, 173, and 181 ms, respectively. These data are shown in Fig 3b. Pairwise comparisons revealed significantly greater RT in the 0 (204 ms) compared to 90 degree offset conditions (173 ms; p<0.001) (IOR = 31 ms), while there was no significant difference in RT between the 90 (173 ms) and 180 degree offset conditions (181 ms; p = 0.125) (OFE = -8 ms). The interaction between target location and cue-target offset was not significant (F(3.29, 55.9) = 2.11, MSE = 1169, p = 0.104).

### E3: Cue-Manual Condition

In the 4 (target location; down, left, right, up) by 3 (cue-target offset; 0, 90, 180) repeated measures ANOVA, the main effect of target location was not significant (F(3,54) = 1.22, MSE = 641, p = 0.311). There was a significant main effect of cue-target offset (F (2, 36) = 4.65, MSE = 550, p = 0.016): mean RTs for 0, 90 and 180-degree offsets were 258, 248, and 249 ms, respectively. These data are presented in Fig 3c. Pairwise comparisons revealed significantly greater RT for the 0 degree (258 ms) as compared to 90 degree cue-target offset condition (248 ms; p = 0.046) (IOR = 10 ms), while there was no significant difference between the 90 (248 ms) and 180-degree cue-target offset conditions (249 ms; p = 1.0) (OFE = -1 ms). The interaction between target location and cue-target offset was not significant (F(6, 108) = 1.51, MSE = 557, p = 0.182).

### E4: Manual-Manual Condition

In the 4 (target location; down, left, right, up) by 3 (cue-target offset; 0, 90, 180) repeated measures ANOVA, the main effect of target location on RT was not significant (F (3,51) = 0.177, MSE = 535, p = 0.911). There was a significant main effect of cue-target offset (F(2,34) = 9.077, MSE = 517, p = 0.001): mean RT for 0, 90 and 180-degree offsets were 233, 222, and 218 ms, respectively. These data are presented in Fig 3d. Pairwise comparisons revealed significantly greater RT for the 0 degree (233 ms) compared to 90 degree offset condition (222 ms; p = 0.046) (IOR = 11 ms), while there was no significant difference between the 90 degree (222 ms) and 180-degree cue-target offset conditions (218 ms; p = 1.0) (OFE = 4 ms). The interaction between target location and cue-target offset was not significant (F (3.30, 56.1) = 1.05, MSE = 897, p = 0.384).

## Discussion

**Fig 4:**
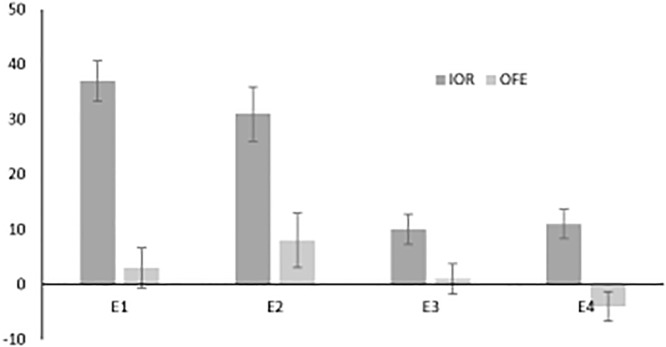
Results from all four experiments. IOR = cued minus orthogonal (+/-90°); OFE = orthogonal (+/-90°) minus uncued (see Fig 1).

The results from our 4 experiments are summarized in Fig 4 where IOR and the OFE are plotted separately for each experiment. We will begin by reviewing the findings from the 4 experiments and relating these to the literature and our original hypotheses, starting with Experiment 3 which used the methodology (ignored peripheral cue followed by peripheral target calling for a manual response) that is most common in the literature that has contrasted the inhibition of return with the attentional momentum mechanisms. Here we replicated the pattern reported by Machado & Rafal [7]: significant IOR (10 ms) and non-significant (-1 ms) OFE. Hence, when eye movements are controlled and trials with untoward eye movements are excluded (as in these two studies) there is no evidence for attentional momentum in a cue-target paradigm with manual responses.

A previous study contrasted these two mechanisms using target-target methods comparable to those employed in Experiment 4, yielding results which are remarkably similar to those reported in the current study [8]. In that study, participants made saccadic or manual responses (separate blocks) to two successive peripheral targets, separated by a central cue-back [8]. Results indicated the presence of reliable IOR (9 ms and 16 ms, for manual and saccadic responses, respectively), but not OFE (6 ms and -1.5 ms for manual and saccadic responses, respectively) (Table 4 in [8]).

In another study [9], participants made keypress responses to a series of targets presented at one of four peripheral locations. Results indicated a robust inhibitory effect at all inter-stimulus intervals investigated. The authors also reported a much smaller opposite facilitation effect, but only for some participants, and only at shorter inter-stimulus intervals [9]. Unlike the present investigation, eye movements were not monitored.

Essentially the results of our E4 were identical to those of E3 (a significant 11 ms of IOR and a non-significant 4 ms of OFE). These findings (like those of Machado and Rafal [7]) are in accord with the Snyder et al. [6] suggestion that maybe occasional cue- or target-elicited eye movements are responsible for the occasional evidence of OFEs. The remaining experiments directly test this idea by requiring eye movements to targets alone (Experiment 1) or to both cues and targets (Experiment 2). In both of these experiments IOR was significant (37 and 31 ms, in the cue-saccade and saccade-saccade experiments, respectively) while the OFE was not only not significant but also in the wrong direction (-3 and -8 ms, respectively). These results strongly suggest that the sporadic observation of OFEs in this literature are not due to the planning and execution of eye movements.

In summary, whereas RTs were significantly slower in the 0 degree offset condition compared to the 90 degree offset condition in all four experiments (i.e., IOR), in no experiment did we find significantly faster RTs to targets opposite from the cued location (180 degree offset condition) compared to the 90 degree offset condition. Hence, the results from all four experiments confirm the predictions made by an inhibitory gradient account, and are inconsistent with those made by attentional momentum. Let us return to the speculative conclusion offered by Snyder et al. [6] that the sporadic finding of OFEs in the literature might be “…an artifact of eye movements or idiosyncratic attentional strategies carried out by participants.” The present experiments rule out the contribution of eye movements per se to these sporadic findings. Hence, until researchers can demonstrate experimental control over the production of OFEs, we would endorse Snyder et al.'s [6] second suggestion and attribute OFEs to as yet unidentified "idiosyncratic attentional strategies" or alternatively OFEs might be a spurious observation.
